# Regulation of immune cell function by short-chain fatty acids

**DOI:** 10.1038/cti.2016.17

**Published:** 2016-04-22

**Authors:** Renan Corrêa-Oliveira, José Luís Fachi, Aline Vieira, Fabio Takeo Sato, Marco Aurélio R Vinolo

**Affiliations:** 1 Department of Genetics, Evolution and Bioagents, Institute of Biology, State University of Campinas, Campinas, São Paulo, Brazil

## Abstract

Short-chain fatty acids (SCFAs) are bacterial fermentation products, which are chemically composed by a carboxylic acid moiety and a small hydrocarbon chain. Among them, acetic, propionic and butyric acids are the most studied, presenting, respectively, two, three and four carbons in their chemical structure. These metabolites are found in high concentrations in the intestinal tract, from where they are uptaken by intestinal epithelial cells (IECs). The SCFAs are partially used as a source of ATP by these cells. In addition, these molecules act as a link between the microbiota and the immune system by modulating different aspects of IECs and leukocytes development, survival and function through activation of G protein coupled receptors (FFAR2, FFAR3, GPR109a and Olfr78) and by modulation of the activity of enzymes and transcription factors including the histone acetyltransferase and deacetylase and the hypoxia-inducible factor. Considering that, it is not a surprise, the fact that these molecules and/or their targets are suggested to have an important role in the maintenance of intestinal homeostasis and that changes in components of this system are associated with pathological conditions including inflammatory bowel disease, obesity and others. The aim of this review is to present a clear and updated description of the effects of the SCFAs derived from bacteria on host immune system, as well as the molecular mechanisms involved on them.

Colonization of the skin, oral cavity, gastrointestinal, genital and respiratory tracts by bacteria, viruses and fungi, microorganims collectively referred as microbiota, is important for the proper functioning of several physiological processes including host tissues development, nutrient absorption and metabolism.^1, 2^ Microbiota–host interaction is also essential for the proper function of the immune system. The development of immune cells, production of effector soluble molecules such as antibodies, antimicrobial peptides and the function of several components of host defense are modulated by the microbiota.^3^ Taking this into account, it is not surprising that the loss of this balance between host immune system and microbiota components is associated with the development of infections and inflammatory diseases such as periodontal disease, bacterial vaginosis, inflammatory bowel disease, rheumatoid arthritis and obesity.^4, 5, 6^

Despite the advances in this field, the molecular mechanisms that account for the interaction microbiota-immune system are not completely understood. Microbial-associated molecular patterns including lipopolysaccharide and peptidoglycans, metabolic molecules including lactic acid and short-chain fatty acids (SCFAs), ATP and others were described to play a role in this context.^3, 7, 8^ However, it has been arduous to establish a relation between microbiota-derived molecules, their host molecular targets and effects on normal and pathological conditions since, among other factors, it is difficult to distinguish their individual contribution to this multicomponent and complex system.

One class of molecules that acts as a link between the microbiota and the immune system is the SCFAs. These are bacterial fermentation products, which are chemically composed by a carboxylic acid moiety and a small hydrocarbon chain. The most common and most studied of them are acetic, propionic and butyric acids, which have, respectively, two, three and four carbons in their chemical structure.

The SCFAs are found in high concentrations in the intestinal tract, where there is a dense population of bacteria that metabolizes indigestible saccharides and, to a lesser extend other molecules, and release the SCFAs as end products of the fermentation process. Concentrations ranging from 70 to 140 mm in proximal colon and 20 to 70 mm in the distal colon have been described for the SCFAs with a predominance of acetate in comparison to the other SCFAs.^9^ In addition, SCFAs are also present in the oral cavity (from 6 to 38 mm of acetate, 1 to 13 mm of propionate and 0 to 5 mm of butyrate) and female genital tract (acetate concentrations may reach 120 mm in the lower genital tract), where their concentrations vary depending on the presence or not of infection/inflammation.^10, 11^

The SCFAs are known to modify several cellular processes including gene expression, chemotaxis, differentiation, proliferation and apoptosis. Signaling pathways such as activation of G protein coupled receptors (GPCRs), inhibition of histone deacetylases (HDACs) and stimulation of histone acetyltransferase activity, among other pathways including the recently described stabilization of the hypoxia-inducible factor (HIF) are implicated in their effects (Figure 1).^12, 13, 14, 15^ SCFAs activate at least four different GPCRs: the free fatty acid receptors (FFAR)-2 and -3, which are also known as GPR43 and GPR41, respectively, the niacin/butyrate receptor GPR109a (also known as HCA2) and the olfatory receptor (Olfr)-78.^16, 17^ These receptors show distinct patterns of expression and they have been partially associated with the effects of the SCFAs on leukocytes and intestinal epithelial cells (IECs), as described at the next sections. More details on the molecular mechanisms and on the effects of SCFAs in other tissues can be found in different reviews at the literature.^14, 18^

Our aim with this review is to present a clear and updated description of the effects of the SCFAs derived from bacteria on host tissues, as well as the molecular mechanisms involved on them. For this, we used mainly studies published in the last 5 years and focused in the interaction between these molecules and immune cells.

## SCFAs interactions with innate mechanisms of defense

The epithelial barriers and the components of innate immune system are important mechanisms of defense against infection. They act with the aim of blocking the entrance of microorganisms and, once they invade the body, rapidly responding to them. In addition, these mechanisms are regulated by adaptive immune components and constitute an essential effector mechanism of this other branch of the immune system.^19^

## Epithelial cells

The epithelial barrier is part of the host defense against microorganisms.^19^ Epithelial cells not only constitute a physical barrier to the entrance of microorganisms, but they are also an active component of the host defense by sensing pathogenic microorganisms or their molecules such as toxins, and responding to them, as shown for IECs.^19^ These cells are source of mucin and antimicrobial peptides that are secreted to the intestinal lumen, as well as immune mediators including cytokines and chemokines, which recruit and regulate the differentiation and activation of immune cells.^19^

As previously described, SCFAs are found in high concentrations in the intestine, where they are in close contact with the IECs. These cells uptake SCFAs through passive (mainly, the non-ionized form) and active mechanisms. Transporters such as the monocarboxylate transporter-1 (Slc16a1) and the sodium-dependent monocarboxylate transporter-1 (Slc5a8) are part of these latter mechanisms responsible for the passage of these bacterial metabolites from the intestinal lumen to the cytosol of IECs, where they are partially used as a source of ATP.^18^ In this context, IECs metabolize most of the absorbed butyrate, while propionate is largely taken up by the liver and acetate reaches the systemic circulation at higher concentrations.^18^ The relevance of SCFAs as energy supply for the colonic epithelium has been demonstrated in different studies. Donohoe *et al.*^20^ exemplifies this concept. These authors showed that colonocytes from germ-free mice present an energy-deprived state and that after their colonization with butyrate-producing bacteria *in vivo* or the addition of butyrate *in vitro* to isolated germ-free colonocytes, it was possible to revert these metabolic alterations leading to an increment in the oxidative phosphorylation and reduction of the autophagy in these cells.^20^

Taking into account the fact that SCFAs are important energetic substrates for epithelial cells, the confirmation that they are regulators of their proliferation was already expected.^12^ Interestingly, SCFAs, mainly butyrate, present different effects on the growth of normal and tumoral colonocytes. Butyrate inhibits the growth of cancerous colonic cells, but not of normal colonocytes and, depending on the concentration, it actually increases the proliferation of this latter cell type.^12, 21^ The mechanism proposed for this difference in susceptibility to butyrate involves the so-called Warburg effect. In brief, cancerous cells present a metabolic shift from oxidative metabolism to glycolysis with lactic acid formation; this impairs the metabolization of butyrate leading to its accumulation inside the cells, favoring the inhibition of HDACs, a mechanism involved in the suppression of cells growth.^12^

Butyrate, and possibly the other SCFAs, play a role in the homeostasis of intestinal epithelium through their effects on colonocytes proliferation and apoptosis. Butyrate may induce colonocyte proliferation at the cript of the colon, where its concentration is lower, while it shows a pro-apoptotic effect close to the lumen, where its concentration increases, inducing colonocyte exfoliation, contributing to the normal turnover of cells in this tissue.^12^ Possibly other effects of SCFAs in the colon also contribute to their role in the homeostasis of this tissue including their capacity to induce the production of IL-18, as described later in this review.

Studies in the literature indicated that SCFAs improve some of the immune defensive functions of the intestinal epithelium. In this regard, Raqib *et al.*^22^ showed that butyrate increases the expression of antimicrobial peptides (LL-37 and CAP-18) by epithelial cells of the large intestine in rabbits and humans. An increment in the cathelicidin LL-37, but not in other antimicrobial peptides such as human beta defensins 1 and 3, was observed in humans after treatment with sodium butyrate enema.^23^

Another study performed with IECs, in this case from pigs, found that butyrate increases the expression of β-defensins and cathelicidins, an effect also observed for acetate and propionate, and extended to porcine macrophages.^24^ Although the mechanism behind these effects of the SCFAs is not completely known, the inhibition of HDAC activity and consequently the modification in the transcription of genes responsible for the synthesis of the antimicrobial peptides is implicated.^25^ Interestingly, some of these studies have found beneficial effects of administration of SCFAs in humans, by reducing the inflammation and increasing the production of LL-37 in patients with shigellosis, and animals (reduction in *Salmonella enteritidis* load in chickens)^23, 25^ suggesting their use for the prevention of enteric infections.

Kim *et al.*^26^ have shown that SCFAs increase the production of cytokines and chemokines including TNF-α, IL-6, CXCL1 and CXCL10 by colonic epithelial cells *in vitro*. This effect was not observed in cells pre-treated with pertussis toxin (an inhibitor of G_0/i_) or in FFAR2- or FFAR3-deficient cells, indicating the involvement of these GPCRs. *In vivo*, the inflammatory response presented by FFAR2- and FFAR3-knockout mice after ethanol-induced breach, trinitrobenzene sulfonic acid treatment or *Citrobacter rodentium* infection was abnormally low. This impairment of the immune response was associated with the absence of SCFAs signals through FFAR2 or FFAR3 in epithelial cells and a consequent reduction in the production of inflammatory mediators, which are necessary for an adequate recruitment of leukocytes and activation of T lymphocytes in the colon in response to inflammatory stimuli.^26^

Other studies reported that SCFAs modify the production of cytokines by IECs. The activation of FFAR2 and GPR109a by SCFAs in IECs increased the production of IL-18, a key cytokine for the repair and maintenance of the epithelial integrity.^27, 28^ Macia *et al.*^27^ demonstrated that after the binding of SCFAs to these GPCRs, there is a membrane hyperpolarization and K^+^ efflux, leading to activation of NLRP3, which promotes the conversion of pro-IL-18 to IL-18. In agreement with a previous study,^7^ the authors of this study found a protective effect of SCFAs in a colitis model, an effect that was associated with the activation of NLRP3 and regulation of inflammation.^27^

SCFAs can also inhibit the production of some cytokines and chemokines by epithelial cells. Iraporda *et al.*^29^ found that SCFAs (mainly, butyrate and propionate) reduce the expression of CXCL8 and CCL20 by Caco-2 cells *in vitro* in response to flagellin stimulation, an effect that was not related to GPCRs activation. The inhibitory effect of the SCFAs on CXCL8, an important neutrophil chemoattractant, was also observed in other IECs cell lines and was associated with the inhibition of HDAC activity.^30^

SCFAs also act on the epithelial barrier function itself. In this context, these bacterial metabolites not only increase the production of IL-18, a cytokine that contributes to the intestinal epithelium homeostasis, but also act by other mechanisms including stabilization of the HIF.^15^ SCFAs increase oxygen consumption by IECs that leads to a reduction in oxygen tension, resulting in a stabilization of HIF.^15^ In this study, the reduction of number and diversity of intestinal microbiota components by antimicrobial agents reduced the expression of HIF-1, which was restored after supplementation with tributyrin (a pro-drug of butyrate) administration. It is worth mentioning that HIF is a transcription factor directly involved in the maintenance of tissue integrity since, among other effects, it regulates the production of antimicrobial peptides and intestinal epithelial tight junctions.^31, 32^

Taking together, these studies indicate that SCFAs are essential for the maintenance of intestinal epithelium physiology both by regulating the cellular turnover and barrier functions, and for modulating the response after inflammatory/infectious stimulation. In this latter condition, however, it is not clear why in some cases these bacterial metabolites present a pro-inflammatory and prompt the immune responses,^26^ whereas in others they have the opposite effect.^28^ The fact that different molecular mechanisms are activated by them on a variety of cell types might partially account for these differences.

## Neutrophils

Another important component of the innate immune defenses is the neutrophil. These cells are normally the first to arrive at the site of inflammation, where they mount a response to infectious agents and produce cytokines that initially orchestrate the subsequent recruitment and activation of other cells including macrophages and even more neutrophils.

SCFAs interact with neutrophils and modulate their recruitment, effector function and survival at different tissues (reviewed in Rodrigues *et al.*^33^). These bacterial metabolites alter neutrophils recruitment by their ability to regulate the production of inflammatory mediators including cytokines that activate endothelial cells such as TNF-α and IL-17 and neutrophil-chemoattractants such as CXCL1 and CXCL8 by different cells.^26, 28, 29, 30, 34, 35^ In addition, SCFAs activation of FFAR2 in neutrophils has been shown to induce their chemotaxis.^7, 36, 37^

Studies using human and rodent neutrophils (in this latter case, elicited cells) have found that SCFAs modify their production of inflammatory cytokines. Tedelind *et al.*^38^ and our group^35^ described an inhibitory effect on the TNF-α production by these cells in the presence of toll-like receptor (TLRs) agonists and SCFAs. A reduction in the activation of the transcription factor NF-κB, which is associated with inhibition of HDAC activity, may account for this effect.^35^ However, another recent study described an increase in the production of CXCL8 by human neutrophils when incubated with a TLR2 agonist and SCFAs, indicating that the effect of these bacterial metabolites may depend on the stimuli and state of activation of the cells.^39^ Other neutrophil functions including their capacity to phagocytose particles and microorganisms, to produce and release toxic molecules such as reactive oxygen species and nitric oxygen (NO) and their survival may also be modified by these bacterial metabolites (revised in Rodrigues *et al.*^33^).

## Monocytes and macrophages

It is obvious that not only the epithelial cells or neutrophils are modulated by the SCFAs. Many studies have been published in the last few years using different experimental approaches to show that monocytes and macrophages are also an important target of these bacterial metabolites. According to Cox *et al.*,^40^ the presence of SCFAs leads to anti-inflammatory effects by regulating the production of cytokines and prostaglandin E_2_ (PGE_2_) by human monocytes. In this study, the authors cultivated human monocytes *in vitro* with or without the SCFAs, pointing out that the presence of these fatty acids enhanced the production of PGE_2_, showing a synergistic result when incubated together with LPS. This result is specifically related to PGE_2_, since other lipid mediators as PGI_2_, LTB_4_, and TXB_2_ were tested, but no effect was observed. Regarding the cytokine profile, there was a reduction of IL-10 caused by the SCFAs when the cells were stimulated with LPS, as well as a dose-dependent inhibition of CCL2 production (with and without LPS). Similar results were obtained in peripheral blood mononuclear cells with an additional result of inhibition in the production of LPS-induced TNF-α and IFN-γ.^40^ Contrary to these findings, another study described a pro-inflammatory (increase of IL-1β, IL-6 and CXCL8/IL-8) effect of SCFAs either alone or, in some cases, combined with TLR agonists, in human peripheral blood mononuclear cells.^39^

As demonstrated above, the scientific literature on the SCFAs shows a lot of divergences. These compounds can act as pro- or anti-inflammatory molecules depending on the cell type that is studied and on the conditions, environment and type of stimulation. Bailón *et al.*^41^ hypothesized that the effects of the SCFAs, especially butyrate, might depend on the states of differentiation and proliferation of the immune cells. To test that, the authors incubated murine bone marrow-derived macrophages with butyrate, showing a dose-dependent inhibition of their proliferation. This effect was also seen in T lymphocytes, although only in this latter cell type a suppression of activation and induction of apoptosis were observed with the treatment. The authors repeated the experiment, but using RAW264.7 murine macrophage-like cells, an immortalized cell line that does not require stopping proliferation before activation. By incubating these cells with butyrate, the authors found results similar to those observed in T cells (and, consequently, divergent from the bone marrow-derived macrophage results), indicating that butyrate leads to an inhibition of the cells that present high proliferative rates during activation (T cells), while apparently it is unable to inhibit the cells that are not proliferating during their activation (tissue macrophages).^41^

The murine macrophage cell line RAW264.7 was also studied by Liu *et al.* ^42^ In this study, cells were incubated with LPS combined with different concentrations of the SCFAs and, even though there was no effect in the cellular viability by the MTT test, a significant reduction in the NO production was observed. The presence of SCFAs also decreased the LPS-induced production of TNF-α, IL-1β, IL-6, although, in the meantime, reinforced the LPS-induced production of IL-10. Interestingly, they also demonstrated that acetate is able to prevent the LPS-induced p65 protein translocation to the nucleus, indicating that the suppression of NF-κB intracellular signaling pathway might be important for the effect of SCFAs. Our group also reported an inhibitory effect of butyrate on TNF-α production by RAW264.7 cells stimulated with palmitic acid or LPS.^43^ Similar results were obtained in the presence of a potent pan-inhibitor of HDACs indicating the involvement of this molecular pathway.^43^

Another study, performed with RAW264.7 cells and rat thioglycolate-elicited macrophages, showed that butyrate inhibits the LPS-induced migration of these cells.^44^ Based on their findings and previous works, the authors proposed a model to explain this effect. LPS binds to the TLR4 on these cells, leading to the activation of NF-κB, which targets iNOS to upregulate Src (a nonreceptor tyrosine kinase) and ultimately activate FAK (focal adhesion kinase), a kinase that interacts with the extracellular matrix and the integrin signaling, and is important for the macrophage migration. In this context, butyrate acts impairing the NF-κB activation, consequently abolishing the increase of Src/FAK and so, reducing the motility of these cells.^44^ More recently, Chang *et al.*^45^ demonstrated the anti-inflammatory effect of butyrate in bone marrow-derived macrophages (a reduction of NO, IL-6 and IL-12p40, but no alteration in the TNF-α or MCP-1/CCL2 was observed in cells stimulated with LPS in the presence of butyrate, but not with propionate or acetate). Similar results were obtained with macrophages isolated from the colonic lamina propria both when the cells were incubated *in vitro* with a combination of butyrate and LPS, and when this SCFA was given orally to the animals. The authors showed that butyrate acts through inhibition of the HDACs. Chromatin immunoprecipitation analyses revealed an increase of the histone 3 lysine 9 acetylation at the promoter regions of the genes *Nos2*, *Il2* and *Il12b*, but not *Tnfa*. In this study, even though butyrate acted as an anti-inflammatory molecule, its presence (given to the mice in the drinking water) did not result in an improvement of resolution in colitis model. Conflicting results regarding the effect of SCFAs in colitis were reported by different groups indicating that differences in the protocol of treatment, model of colitis or other factors including type of diet may be affecting the disease outcome.^7, 28, 46^

Regarding the production of host defense peptides, Zeng *et al.*,^24^ as cited above, showed that butyrate induces the production of some of these peptides in a dose-dependent manner in porcine 3D4/31 lung alveolar macrophages, as well as in primary monocytes, and IPEC-J2 epithelial cells. Besides that, similar results have been described in humans, rabbits and cattle, but apparently butyrate is not a host defense peptides inducer in mice.^22, 47, 48^ Sunkara *et al.*,^49^ using chicken HD11 macrophage cells and chicken primary monocytes, showed that the presence of butyrate enhances the expression of many host defense peptides genes such as Avian β-defensin 9 (*AvBD9*), *cathelicidin B1*, *AvBD3*, *AvBD4*, *AvBD8*, *AvBD10* and *AvBD14*, helping to suppress bacterial growth. Moreover, by given the chickens butyrate in the drinking water, there was a reduced colonization of *Salmonella enteriditis* in the cecum. However, different than what was described for mammalian cells, the presence of butyrate did not alter the production of cytokines as IL-1β, IL-8 and IL-12p40 by avian cells. Another important aspect is that no changes in the phagocytic capacity or the oxidative burst of HD11 cells were observed after the treatment with different concentrations of butyrate.^49^ A more recent paper of this group indicated that acetate and propionate also present these effects.^25^ Together, these articles demonstrated that SCFAs modulate macrophage responses.

## Dendritic cells (DCs)

Millard *et al.*^50^ investigated whether the SCFAs, specifically butyrate, could affect macrophages and DCs differentiation and functions. Using a non-cytotoxic concentration of butyrate, these authors showed that its addition into a culture of human peripheral blood monocytes with the proper growth factors to macrophages or DCs differentiation (GM-CSF only or GM-CSF and IL-14, respectively) caused important modifications in their phenotypic differentiation, leading to changes in the capacity of these cells to capture antigens (confirmed by tests of phagocytic capacity). Moreover, they also showed that not only butyrate inhibited the maturation of the DCs when these cells were incubated with different inducers as TNF-α+PGE_2_, LPS or even TNF-α+IL-1β, but it also altered the production of some cytokines as IL-10 and IL-12 by this cell type. As predicted, DCs pre-treated with butyrate showed a lower capacity to stimulate T cells.^50^

A delay in human DCs maturation, characterized by an inhibition in dendrite formation and expression of surface markers as CD80, CD83, CD1a and MHC class II molecules (which are highly expressed on mature DCs), was observed in the presence of butyrate.^42^ DCs treated with butyrate had a lower capacity of stimulating T cells and showed a reduction in the production of pro-inflammatory cytokines as IL-12p40 and IFN-γ. In contrast, DCs treated with butyrate released much higher amounts of the anti-inflammatory cytokine IL-10.^42^ Other studies have also described a reduction in the expression of surface markers associated with the maturation of DCs such as CD40, CD80 and CD86 in cells incubated with butyrate or the other SCFAs.^51, 52^ In addition, a study reported that butyrate increases the production of IL-23 by DCs, an effect with important implications for the polarization of T cells.^51^

Singh *et al.*^53^ investigated the mechanisms by which the SCFAs regulate DCs development and function. In this study, butyrate suppressed DCs development, but not their functional maturation after LPS stimulation. This effect on DCs development was also observed in cells incubated with propionate, but not acetate.^53^ According to the authors, the ability of these compounds to inhibit the HDACs, thus suppressing the expression of important transcription factors for DC development such as *PU.1* and *RelB*, is involved in their effects on this cell type. Interestingly, this study also revealed that these modulations are in fact dependent on the butyrate-transporter Slc5a8,^53^ whose role in the effects of SCFAs in DCs and other immune cells was explored in more details in another study.^54^

In summary, it is now well-established that SCFAs modulate different aspects of innate immune response, even though there are still some controversies in the literature. The actions of SCFAs on innate immunity also affect the activation (SCFAs have been shown to impair DCs development affecting their ability to stimulate T lymphocytes) and the effector response of the components of the adaptive immunity. In addition, direct effects of SCFAs on T lymphocytes have already been reported, as described in the next section of this review.

## Impact of the SCFAs on the adaptive immune system

The SCFAs modulate T-lymphocytes activation and effector responses. Several studies have shown that SCFAs, in general, induce a T-lymphocyte tolerogenic profile, which depends on their actions in the activation/differentiation of DCs and macrophages as well as direct effects on lymphocytes.

Gurav *et al.*^54^ have found that DCs treated *in vitro* with butyrate and, to a lesser extend with propionate, present an increment in the expression of indoleamine 2,3-dioxygenase 1 and aldehyde dehydrogenase 1A2. These enzymes attenuate the immune activation through triptophan depletion (IDO) and generation of retinoic acid (aldehyde dehydrogenase 1A2), a molecule with immunesuppressive properties. These effects, together with other SCFAs actions on DCs, potentiate the ability of these cells to convert naïve T cells into FoxP3^+^ regulatory T cells (Tregs) and to suppress their conversion into pro-inflammatory T cells (IFN-γ^+^ T cells).^54^ In accordance with Singh *et al.,*^53^ the authors indicated the involvement of the Slc5a8 transporter and, possibly, the inhibition of HDACs activity, in the effects of SCFAs on DCs. ^54^

Singh *et al.*^28^ demonstrated that butyrate activation of GPR109a in macrophages and DCs is essential for maintaining the balance between pro- and anti-inflammatory CD4^+^ T cells. In this study, the authors found that GPR109a-deficient mice (GPR109a KO) have a reduction in CD4^+^ T-cells-producing IL-10 and an increase of IL-17-producing T cells. This phenotype was associated with the absence of GPR109a signaling activated, possibly, by butyrate derived from the microbiota, in the GPR109a KO animals. Macrophages and DCs incubated with butyrate or niacin (other agonist of GPR109a) showed an increase in expression of *l10* and *Aldh1a1*, which contribute for the differentiation of naive T cells to regulatory T lymphocytes. In addition, the authors found, as previously discussed, that butyrate increased the expression of IL-18 in colonic epithelium. Together, these effects of GPR109a agonists may explain why GPR109a KO mice are more susceptible to colitis, intestinal inflammation and carcinogenesis.^28^

SCFAs induce generation of Tregs not only through their effects on DCs, but also by theirs direct interaction with T lymphocytes. In this context, Arpaia *et al.*^55^ demonstrated that butyrate facilitates extra-thymic peripheral polarization to Treg Foxp3^+^ both *in vivo* and *in vitro*. *In vivo*, there was a significant increase in the amount of peripheral regulatory T cells in antibiotic-treated mice that received butyrate in the drinking water, a pattern that was not seen in the thymus or in the colon. Using mice deficient in a Foxp3 enhancer, the conserved noncoding sequence 1 (these animals have an intact thymic differentiation, but present a deficient peripheral generation of Treg cells), the authors showed that this increase in Tregs by SCFAs (propionate and butyrate) was due to the extra-thymic generation of these cells. The proposed mechanism for these effects of SCFAs was the inhibition of HDACs.^55^ Another study has found that the generation of peripheral regulatory T cells in the colon by a class of commensal bacteria that predominates in the intestinal tract, the Clostridia class, is associated with their production of butyrate *in vivo*.^56^ In agreement with Arpaia *et al*,^55^ the mechanism proposed in this study for the Treg-cell-polarizing effect of butyrate was the inhibition of HDACs. The inhibition of this class of enzymes leads to an enhanced histone H3 acetylation in the *locus* of Foxp3 and increases the expression of this transcription factor.^56^ The activation of FFAR2 also seems to play a role in the effect of SCFAs in the Treg generation.^46^

SCFAs can also affect Th1, Th2 and Th17 polarization and activation. Trompette *et al.*^4^ have shown that SCFAs produced in the intestine impair Th2 polarization. The authors found that mice fed with a fiber-enriched diet (increased circulating levels of SCFAs) are more resistant against allergic processes in the lungs. The opposite phenotype was observed in animals fed with a low-fiber diet. This effect was associated with the ability of SCFAs (mainly, propionate) to modify hematopoiesis and increase the generation of precursors of macrophages and DCs with low MHC-II and CD40 expression, and increased phagocytic capacity. In this sense, animals, previously treated with propionate, showed increased recruitment of these cells, which are less effective in reactivating Th2 cells, to lung-draining lymph nodes. Likewise, there were fewer eosinophils, IgE and cytokines such as IL-4 and IL-13 in the lung of these animals. Interestingly, the authors showed that these effects are dependent of FFAR3, but independent of FFAR2. ^4^ In this context, a recent study showed that high-fiber diet or acetate administration protected mice against the development of allergic airways disease.^57^ Interestingly, high-fiber/acetate feeding of pregnant mice also led to a reduction in allergic airways disease development by their adult offspring. This study also provides evidence for a role of diet and acetate production in the development of airway disease in humans.^57^

Nevertheless, Park *et al.*^58^ have demonstrated that acetate, propionate and butyrate enhance the naïve T-cell polarization not only to Tregs, but also promote the generation of Th17 and Th1 effector cells *in vitro.* Interestingly, however, the Th1 and Th17 cells induced *in vitro* in the presence of SCFAs presented a less inflammatory profile *in vivo* in a colitis model. The authors indicated that this is associated with the ability of SCFAs to induce IL-10-producing T cells together with Th1 and Th17 effector T cells. The effect of the SCFAs in the T cells was again associated with the inhibition of HDACs, but in this case mTOR activation was also suggested to participate in the mechanism.^58^ Other study, in which, splenocytes were co-cultured with LPS-stimulated DCs, reported that butyrate treatment *in vitro* caused a significant induction of IL-17 and IL-10. However, in this study, the authors found that oral administration of butyrate increased colitis severity.^51^ SCFAs are also able to modulate T-cells proliferation and apoptosis. A low concentration of butyrate was shown to inhibit the proliferation of both CD4^+^ and CD8^+^ T cells *in vitro*. This study also reported an induction of apoptosis in T cells through a Fas-dependent mechanism.^59^

Taking together, these studies suggest that SCFAs affect the activation and effector function of T cells. However, despite the fact that most of the studies support the idea that SCFAs induce a tolerogenic and anti-inflammatory profile of T cells, evidences also indicate that under some conditions they may induce Th1 and Th17 responses, and, depending on the disease/model and other factors including time, route and concentrations of treatment used, they can both ameliorate or worsen the disease severity.^51, 52, 58, 60^

## Concluding remarks and perspectives in the field

**Figure 2:**
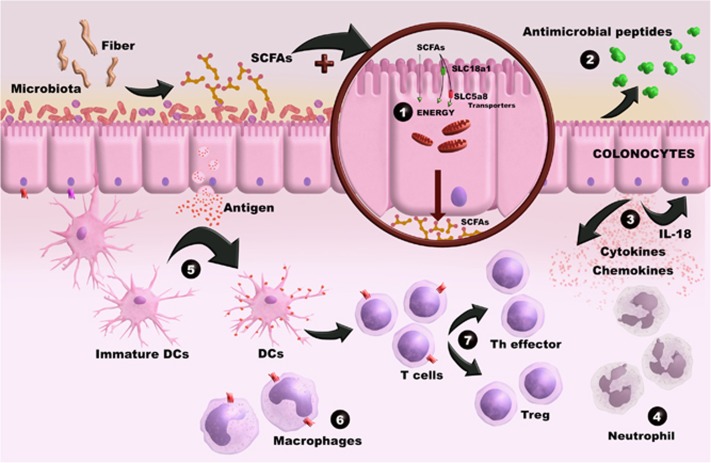
The SCFAs are bacterial fermentation products found in high concentrations in the intestine. These metabolites act as a link between the microbiota and the immune system. IECs uptake SCFAs through passive (mainly, the non-ionized form) and active mechanisms. Once inside the cells, they are partially used as a source of energy (1). In addition, these SCFAs increase the expression of antimicrobial peptides secreted to the external surface by epithelial cells (2) and modulate their production of immune mediators including IL-18, a key cytokine for the repair and maintenance of epithelial integrity, and others cytokines and chemokines (3). SCFAs can also regulate the differentiation, recruitment and activation of immune cells: including neutrophil (4), DCs (5), macrophages (6) and T lymphocytes (7). In this context, SCFAs interact with neutrophils and modulate their recruitment, effector function and survival at the tissues (4). In general, these bacterial metabolites present anti-inflammatory effects including reduction of some pro-inflammatory cytokines such as TNF-α and IL-12 production by macrophages and DCs, and change their capacity to capture antigens and stimulate T cells. In addition, the SCFAs also modulate the proliferation and differentiation of T lymphocytes through direct effects on these cells (for example, inducing the generation of Tregs) (7).

The interaction between microbiota and immune system is bidirectional and involves different components/mechanisms, which are beginning to be identified and understood in physiological and pathological states. SCFAs, as described in this review, are an important link between microbiota and immune system. This interaction involves different molecular mechanisms and cellular targets, as summarized in the Figure 2, and it is essential for the maintanence of intestinal homeostasis and also plays a role in the development of diseases. Despite the advances in the field, several aspects of this interaction remain unclear or need to be studied in more details as indicated by the conflicting results described in the literature.

Our expectation is that this flourishing area of research will impact on our knowledgement on the mechanisms by which diet, microbiota and also other factors influence the functioning of immune system and, consequently, the development of inflammatory and infectious diseases. In this context, it is important to mention that this knowledge will open opportunities for developing novel and more effective therapies for the treatment of chronic inflammatory diseases.

## Figures and Tables

**Figure 1:**
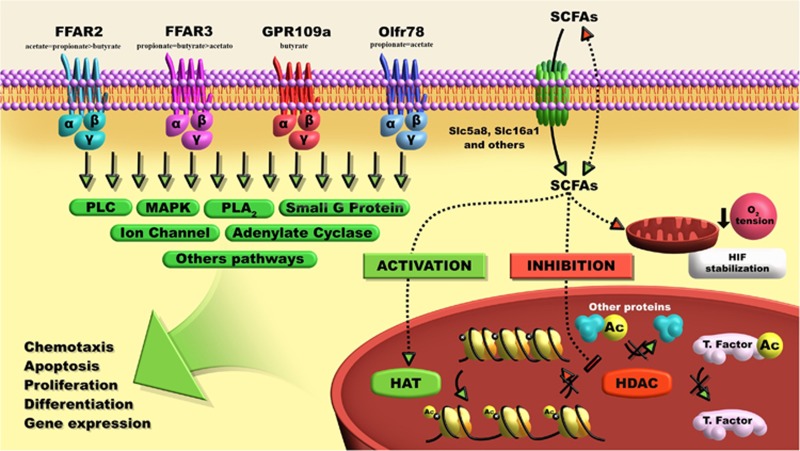
Cellular signaling pathways activated by the short-chain fatty acids. These bacterial metabolites activate membrane receptors called GPCRs (as FFAR2, FFAR3, GPR109a and Olfr78). They are also able to reach the cytoplasm of the cells through transporters (Slc16a1 and Slc5a8) or passive diffusion across the plasma membrane (mainly, the non-ionized form) and they modulate the activity of several enzymes and transcription factors including the HIF, HDACs and histone acetyltransferase (HAT). SCFAs modify several cellular processes including gene expression, chemotaxis, differentiation, proliferation and apoptosis through these mechanisms.
